# Evaluation of the fused deposition modeling and the digital light processing techniques in terms of dimensional accuracy of printing dental models used for the fabrication of clear aligners

**DOI:** 10.1002/cre2.366

**Published:** 2020-11-30

**Authors:** Samer T. Jaber, Mohammad Y. Hajeer, Tarek Z. Khattab, Luai Mahaini

**Affiliations:** ^1^ Department of Orthodontics University of Damascus Dental School Damascus Syria; ^2^ Department of Orthodontics University of Hamah Dental School Hamah Syria

**Keywords:** 3D printed models, digital light processing, fused deposition modeling, orthodontic models

## Abstract

**Objective:**

The objective of this study was to assess the accuracy of physical reproductions of plaster orthodontic study casts fabricated by two different rapid prototyping techniques: Fused Deposition Modeling (FDM) and Digital Light Processing (DLP).

**Materials and methods:**

Twenty pairs of pretreatment plaster models were prepared from randomly selected patients at the Orthodontic Department, University of Damascus Dental School. Twenty‐one reference points were placed on plaster models, followed by scanning and printing of these models using FDM and DLP techniques. Forty measurements were made on these models using a digital caliper. Paired *t* tests were used to detect significant differences in the measurements between the 3D printed replicas and the original plaster models (Gold Standard). Alpha level was adjusted due to the multiplicity of the tests.

**Results:**

The intraclass correlation coefficients for all the comparisons made between the 3D replicas and the gold standard models were greater than 0.80 with ICCs ranging from 0.802 to 0.990 and from 0.853 to 0.990 for the FDM and DLP techniques, respectively. This indicated an excellent agreement. No statistically significant differences could be detected between the 3D‐printed models and their corresponding plaster models. The overall mean difference was −0.11 mm and 0.00 ranging from −0.49 to 0.17 mm and from −0.42 to 0.50 mm, for the FDM and DLP techniques, respectively.

**Conclusion:**

The accuracy of the 3D models produced by the DLP and FDM techniques was acceptable. However, for the fabrication of clear aligners, the optimum fit of the produced plates in the patients' mouths is not completely guaranteed.

## 1 INTRODUCTION

Recent advances in digital dentistry have paved the way for more innovative treatment and treatment planning strategies such as the use of computer‐aided design (CAD) technology in orthodontic practice (Cole et al., 2019). Digital technologies including intraoral digital scanners, three‐dimensional (3D) model scanners, and cone‐beam computed tomography have gained popularity in clinics and labs recently (Hajeer, 2014). Digital impressions taken by intraoral scanners save patients from unpleasant alginate impressions (Burzynski et al., 2018), while also providing orthodontists with an efficient and convenient way to store patient data (hard disk instead of physical space) (Mcguinness & Stephens, 1992; Tancu et al., 2019). However, working without plaster models is not yet regular in the daily practice. Orthodontists prefer physical models to digital models because the former is tangible and more practical in direct communications. Furthermore, the laboratory manufacturing of the orthodontic appliances especially the clear aligners still requires physical models (Cole et al., 2019; Hazeveld et al., 2014; Kim et al., 2018).

Recently, 3D printers facilitate directly fabrication of dental and orthodontic appliances from 3D models. 3D printing is an additive manufacturing process where materials are added layer on layer to produce an object, in contrast with reductive manufacturing in which material is subtracted to produce the object. Rapid prototyping is another term for additive manufacturing (Beguma & Chhedat, 2014; Kim et al., 2018). Several printing technologies exist such as stereolithography apparatus printing (SLA), digital light processing (DLP), Polyjet printing, and fused deposition modeling (FDM). These technologies are all related to the basic principle but with differences mainly in the material and the method used to produce the objects. SLA technique is a rapid prototyping process that utilizes an ultraviolet (UV) laser to cure a liquid polymer into a solid resin (Dietrich et al., 2017; Rebong et al., 2018). DLP technique is similar to stereolithography but it uses visible light‐sensitive resins instead of an ultraviolet laser for curing each layer (Hazeveld et al., 2014). Polyjet printing uses jet heads that spray thousands of photopolymer droplets onto a build platform and solidifying them with a UV light, based on the 3D coordinates given to the jet heads (Brown et al., 2018; Kim et al., 2018). FDM (also known as Fused Filament Fabrication [FFF]) technique consists of a movable head, which deposits a thread of molten medical‐grade acrylonitrile butadiene styrene (ABS) material on the substrate. The build material is heated to 0.5°C above its melting point so that it solidifies about 0.1 s after extrusion and cold welds to the previous layer (Jockusch & Özcan, 2020; Murugesan et al., 2012). Therefore, digital files can be reconstructed and printed into physical models by a large selection of 3D printers with different printing technologies (Brown et al., 2018; Hazeveld et al., 2014; Kim et al., 2018). One of the greatest advantages of the FDM technique is its simplicity and the cheap cost of materials which have made it a reasonable choice for model fabrication in comparison with the relatively more expensive printing technologies such as the DLP technique (Kasparova et al., 2013; Kim et al., 2018). There is a limited number of studies about the accuracy of 3D dental models printed by the FDM technique in comparison with the relatively higher‐cost printing technologies (e.g., the DLP technique). The objective of the study was to assess the accuracy of physical reproductions of plaster orthodontic study casts fabricated by two different rapid prototyping processes: DLP and FDM techniques. The null hypothesis stated that there were no significant differences between the DLP‐ and the FDM‐printed models compared to the original plaster models in terms of accuracy.

## 2 MATERIALS AND METHODS

### 2.1 Setting and models' selection

This study was conducted at the Department of Orthodontics, University of Damascus Dental School and was funded by University of Damascus Postgraduate Research Budget (Ref no: 8305420750DEN). Forty maxillary and mandibular pretreatment plaster models were prepared for the patients referred to the Orthodontic Department at the University of Damascus Dental School. The age of recruited patients ranged between 16 and 25 years; 8 male and 12 female volunteers had Class I (8), Class II (9), and Class III malocclusions (3). Nine of these patients had also crowding in the anterior area. Models had to match the following criteria to be included in the study: (1) Full eruption of all permanent teeth, the second and third molars were not considered; (2) no extracted teeth; (3) normal crown morphology; (4) high‐quality dental casts (i.e., no broken teeth, air bubbles, or voids) which should be trimmed and polished according to the American Board of Orthodontics [ABO] standards).

### 2.2 Inserting reference points and scanning

**FIGURE 1:**
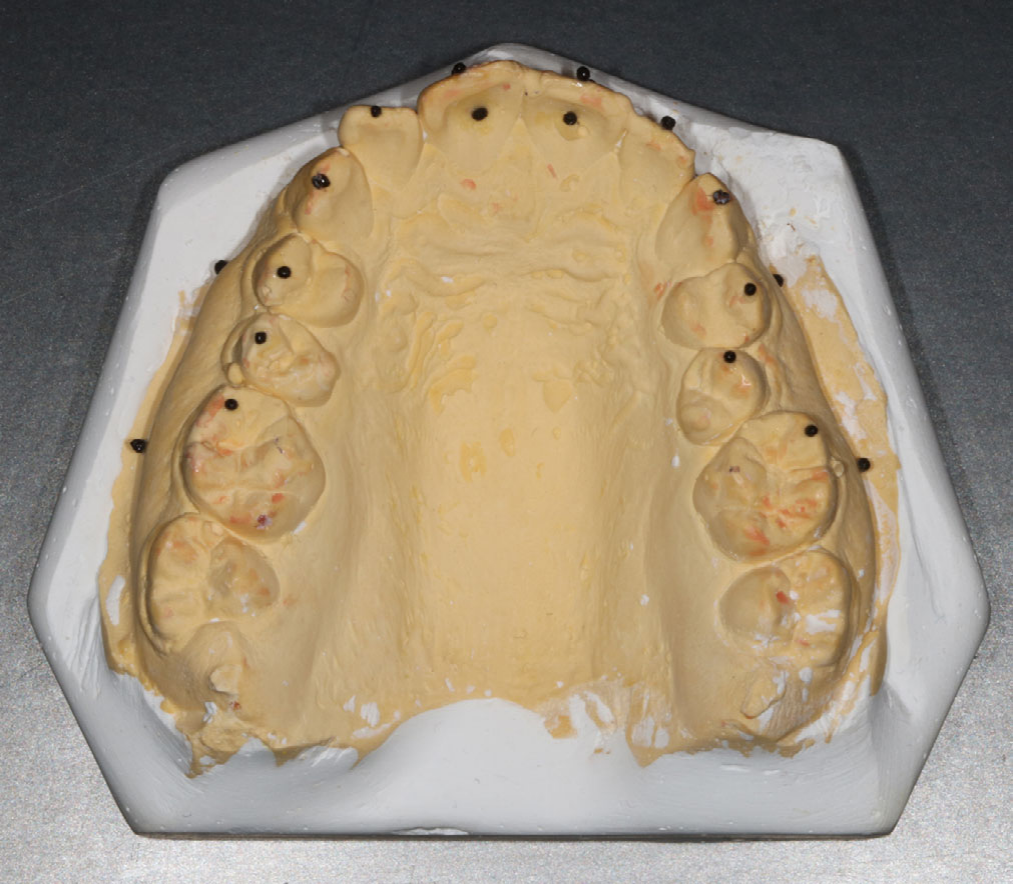
A study model with the reference points attached to it

**FIGURE 2:**
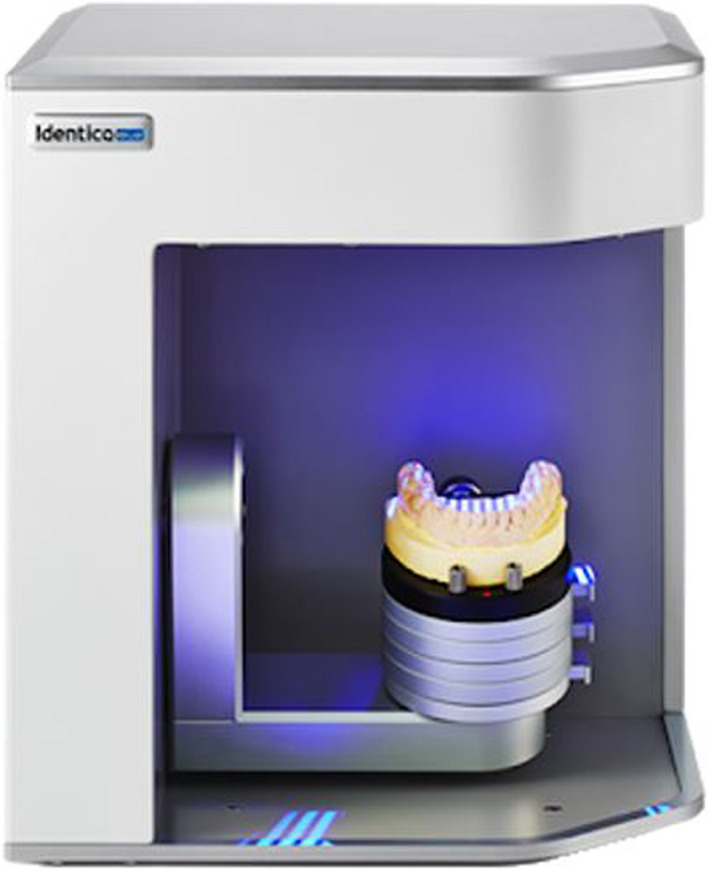
The 3D model scanner used in the study

Twenty‐one reference ball‐shaped markers with a diameter of about 0.5 mm were placed on each model as reference points (or landmarks) for the measuring procedure (Figure 1). Twelve points were placed on the occlusal surfaces of the teeth from the first molar on one side to the first molar on the other side, five points were placed on the buccal gingivae, and four points were placed on the buccal and lingual surfaces of the central incisors. The dental models were scanned using a 3D model scanner (Identica Hybrid; MEDIT, Seoul, Korea) with a precision of ±7 μm (Figure 2). All scanned files were converted into the stereolithographic format (.stl). The “.stl” file format is widely used in 3D printing software to generate information needed to produce 3D models by rapid prototyping processes.

### 2.3 Printing and measuring

**FIGURE 3:**
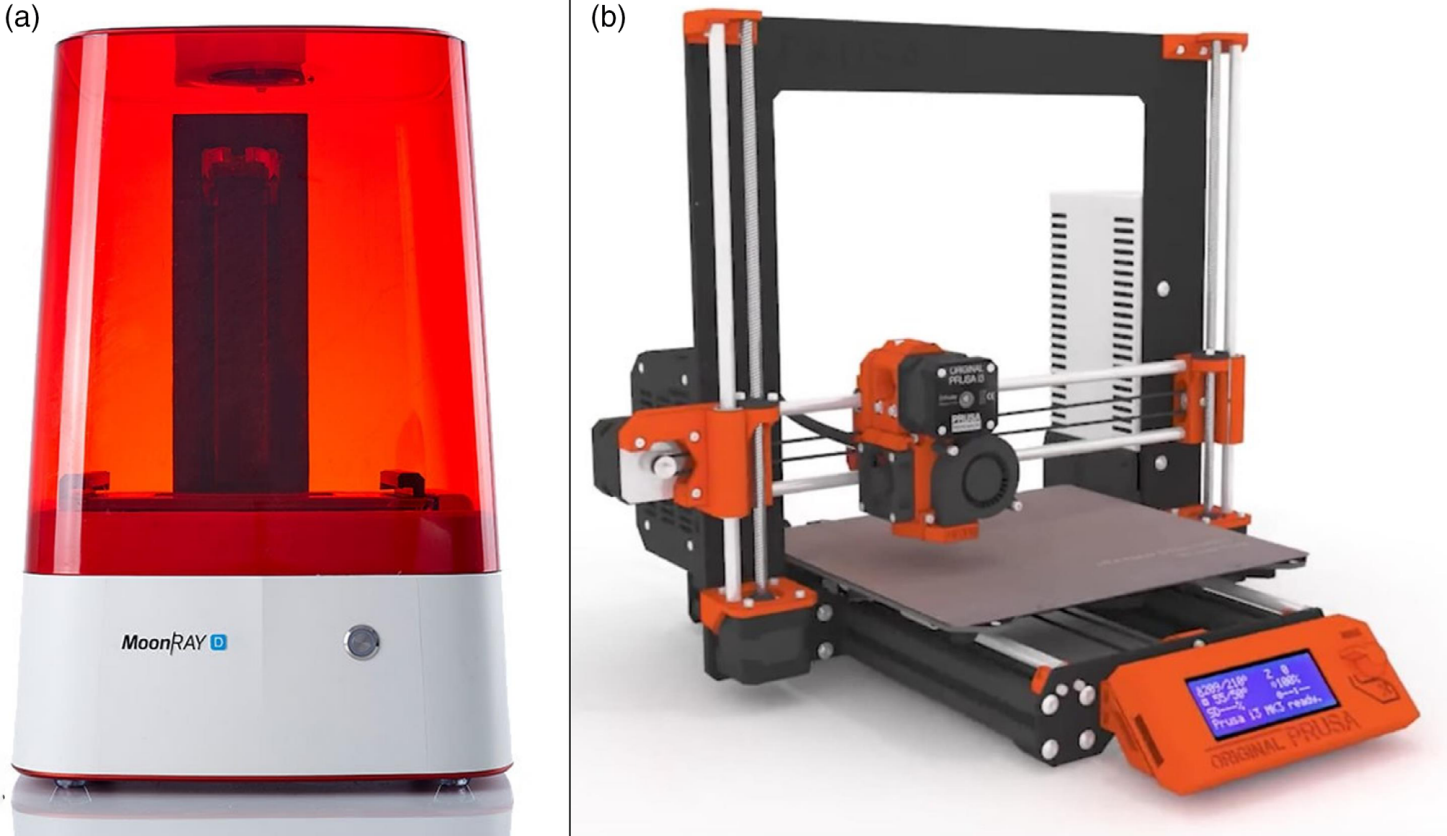
Printers used in the current investigation: The DLP 3D printer (a), The FDM 3D printer (b)

**FIGURE 4:**
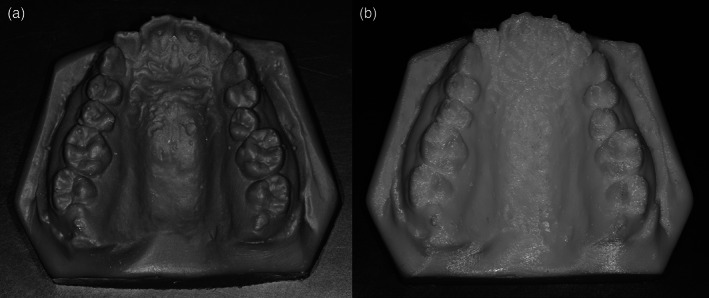
Examples of printed models: using the DLP technique (a), using the FDM technique (b)

Two types of 3D printers were selected (Figure 3), based on the printing technique: DLP technique (Moonray; Sprintray, Los Angeles, CA), and FDM technique (i3 MK3; Prusa, Czech). The reference models were printed using these two printers (Figure 4). Forty inter‐landmark linear measurements were made on each set of models (plaster and replica; 20 measurements for each jaw) by the principal researcher (S.T.J) using a calibrated electronic digital caliper (O4OO‐EEP, Orthopli, Philadelphia, PA) with an accuracy of 0.01 mm. Measurements were categorized into 4 groups: intra‐arch width, inter‐dental, within‐gingival, and tooth thickness measurements. Definitions of the measurements made in the upper and lower models were listed in Tables 1 and 2, respectively.

**TABLE 1:** Definitions of the measurements made in the upper jaw (in mm)

Measurements^a^		Definition
1	UCCW	Distance between the reference points on the cusp tips of the 13, 23
2	UPPM	Distance between the reference points on the buccal cusp tips of 14,24
3	UMMW	Distance between the reference points on the mesio‐buccal cusp tips of 16, 26
4	D16‐15^b^	Distance between the reference point on the mesio‐buccal cusp tip of 16 and the reference point on the buccal cusp tip of 15.
5	D15‐14	Distance between the reference point on the buccal cusp tip of 15 and the reference point on the buccal cusp tip of 14.
6	D14‐13	Distance between the reference point on the buccal cusp tip of 14 and the reference point on the cusp tip of 13.
7	D13‐12	Distance between the reference point on the cusp tip of 13 and the reference point on the middle of the cutting edge of 12.
8	D12x11	Distance between the reference point on the middle of the cutting edge of 12 and the reference point on the middle of the cutting edge of 11.
9	D11‐21	Distance between the reference point on the middle of the cutting edge of 11 and the reference point on the middle of the cutting edge of 21.
10	D21‐22	Distance between the reference point on the middle of the cutting edge of 21 and the reference point on the middle of the cutting edge of 22.
11	D22‐23	Distance between the reference point on the middle of the cutting edge of 22 and the reference point on the cusp tip of 23.
12	D23‐24	Distance between the reference point on the cusp tip of 23 and the reference point on the buccal cusp tip of 24.
13	D24‐25	Distance between the reference point on the buccal cusp tip of 24 and the reference point on the buccal cusp tip of 25.
14	D25‐26	Distance between the reference point on the buccal cusp tip of 25 and the reference point on the mesio‐buccal cusp tip of 26.
15	G16‐14	Distance between the reference point on the muco‐gingival junction of 16 and the reference point on the muco‐gingival junction of 14.
16	G14‐11	Distance between the reference point on the muco‐gingival junction of 14 and the reference point on the muco‐gingival junction of 11.
17	G21‐24	Distance between the reference point on the muco‐gingival junction of 21 and the reference point on the muco‐gingival junction of 24.
18	G24‐26	Distance between the reference point on the muco‐gingival junction of 24 and the reference point on the muco‐gingival junction of 26.
19	U1ThickR	Distance between the reference points on the buccal and palatal surfaces of 11.
20	U1ThickL	Distance between the reference points on the buccal and palatal surfaces of 21.

^a^ From 1 to 3, intra‐arch width measurements; from 4 to 14, inter‐dental measurements; from 15 to 18, within‐gingival measurements; 19 and 20, tooth thickness measurements.

^b^ FDI dental numbering system was used.

**TABLE 2:** Definitions of the measurements made in the lower models (in mm)

Measurements^a^		Definition
21	LCCW	Distance between the reference points on the cusp tips of the 33, 43
22	LPPW	Distance between the reference points on the buccal cusp tips of 34, 44
23	LMMW	Distance between the reference points on the mesio‐buccal cusp tips of 36 46
24	D36‐35^b^	Distance between the reference point on the mesio‐buccal cusp tip of 36 and the reference point on the buccal cusp tip of 35.
25	D35‐34	Distance between the reference point on the buccal cusp tip of 35 and the reference point on the buccal cusp tip of 34.
26	D34‐33	Distance between the reference point on the buccal cusp tip of 34 and the reference point on the cusp tip of 33.
27	D33‐32	Distance between the reference point on the cusp tip of 33 and the reference point on the middle of the cutting edge of 32.
28	D32‐31	Distance between the reference point on the middle of the cutting edge of 32 and the reference point on the middle of the cutting edge of 31.
29	D31‐41	Distance between the reference point on the middle of the cutting edge of 31 and the reference point on the middle of the cutting edge of 41.
30	D41‐42	Distance between the reference point on the middle of the cutting edge of 41 and the reference point on the middle of the cutting edge of 42.
31	D42‐43	Distance between the reference point on the middle of the cutting edge of 42 and the reference point on the cusp tip of 43.
32	D43‐44	Distance between the reference point on the cusp tip of 43 and the reference point on the buccal cusp tip of 44.
33	D44‐45	Distance between the reference point on the buccal cusp tip of 44 and the reference point on the buccal cusp tip of 45.
34	D45‐46	Distance between the reference point on the buccal cusp tip of 45 and the reference point on the mesio‐buccal cusp tip of 46.
35	G36‐34	Distance between the reference point on the muco‐gingival junction of 36 and the reference point on the muco‐gingival junction of 34.
36	G34‐31	Distance between the reference point on the muco‐gingival junction of 34 and the reference point on the muco‐gingival junction of 31.
37	G41‐44	Distance between the reference point on the muco‐gingival junction of 41 and the reference point on the muco‐gingival junction of 44.
38	G44‐46	Distance between the reference point on the muco‐gingival junction of 44 and the reference point on the muco‐gingival junction of 46.
39	L1ThickL	Distance between the reference points on the buccal and lingual surfaces of 31.
40	L1ThickR	Distance between the reference points on the buccal and lingual surfaces of 41.

^a^ From 21 to 23, intra‐arch width measurements; from 24 to 34, inter‐dental measurements; from 35 to 38, within‐gingival measurements; 39 and 40, tooth thickness measurements.

^b^ FDI dental numbering system was used.

Any identification codes on the models were removed or covered, and a study identification code was given to each model by a third person not involved in this research project. Investigator blinding was not possible during the study, as the plaster and printed models produced by each of the tested printers had unique colors and appearances. To avoid any bias during measurement, all models from one printer were measured at once before proceeding to measure the models from the other printer.

### 2.4 Statistical analysis

The data were analyzed using SPSS version 20 (IBM Corporation., Chicago, IL). The Anderson‐Darling test was applied to check the Normality of the distributions. Paired *t* test was used to test for significant differences between the printed models and the gold standard original plaster models. The alpha level was adjusted and set at 0.002 employing Bonferroni's correction (i.e., 20 comparisons made).

### 2.5 Error of the method and assessment of reliability

All the models were re‐measured one‐week after the first measurement. To detect any systematic error paired *t* tests were assessed. No significant differences were found between the repeated measurements (*p* ˃ 0.002). For the intra‐rater reliability, Intra‐class correlation coefficients (ICCs) were used. The ICCs between the two sets of measurements ranged between 0.814 and 0.999 indicating that the intra‐rater reliability was excellent.

## 3 RESULTS

The differences in the measurement made between plaster and resin models on the upper and lower dental arches are shown in Tables 3 and 4 respectively.

**TABLE 3:** Descriptive statistics of the measurements made on the three sets of models (*n* = 20) as well as the mean differences between the two types of printed models and the gold standard plaster models in conjunction with the *p*‐values of statistical testing and the intra‐class correlation coefficients

Measurements	Type	Mean	SE	SD	Mean difference (95% CI)	SE mean difference	*p*‐value^a^	ICC^b^
UCCW	FDM	34.01	0.47	2.11	0.04 (−0.63, 0.72)	0.32	0.893	0.916
	DLP	33.77	0.63	2.82	0.20 (−1.11, 1.50)	0.62	0.755	0.926
UPPM	FDM	40.30	0.64	2.85	−0.24 (−0.49, 0.00)	0.12	0.053	0.990
	DLP	40.00	0.50	2.22	0.48 (−0.37, 1.35)	0.44	0.228	0.980
UMMW	FDM	49.36	0.56	2.50	0.17 (−0.17, 0.51)	0.16	0.314	0.980
	DLP	48.94	0.69	3.10	0.26 (−0.33, 0.85)	0.28	0.366	0.989
D16‐15	FDM	5.89	0.20	0.91	−0.15 (−0.49, 0.20)	0.17	0.394	0.881
	DLP	5.99	0.19	0.84	0.05 (−0.35, 0.45)	0.19	0.804	0.981
D15‐14	FDM	6.86	0.19	0.83	−0.23 (−0.49, 0.02)	0.12	0.072	0.848
	DLP	7.17	0.16	0.72	−0.08 (−0.27, 0.11)	0.09	0.389	0.948
D14‐13	FDM	8.26	0.19	0.85	−0.21 (−0.63, 0.19)	0.20	0.288	0.971
	DLP	8.69	0.22	1.00	−0.21(−0.70, 0.27)	0.23	0.373	0.976
D13‐12	FDM	8.10	0.21	0.92	0.17 (−0.07, 0.40)	0.11	0.149	0.939
	DLP	7.99	0.23	1.02	−0.05 (−0.26, 0.16)	0.10	0.626	0.944
D12‐11	FDM	7.14	0.22	0.96	0.02 (−0.29, 0.34)	0.15	0.880	0.885
	DLP	7.22	0.24	1.06	−0.10 (−0.38, 0.18)	0.14	0.475	0.898
D11‐21	FDM	7.45	0.26	1.16	0.08 (−0.09, 0.25)	0.08	0.340	0.975
	DLP	7.57	0.26	1.15	−0.20 (−0.43, 0.04)	0.11	0.096	0.957
D21‐22	FDM	6.84	0.19	0.83	0.00 (−0.23, 0.23)	0.11	0.989	0.912
	DLP	6.93	0.21	0.95	−0.09 (−0.27, 0.09)	0.09	0.327	0.918
D22‐23	FDM	7.65	0.23	1.03	−0.09 (−0.28, 0.10)	0.09	0.346	0.877
	DLP	8.08	0.22	1.00	0.06 (−0.38, 0.50)	0.21	0.777	0.968
D23‐24	FDM	7.98	0.25	1.11	−0.05 (−0.28, 0.17)	0.11	0.624	0.802
	DLP	8.24	0.24	1.09	−0.22 (−0.51, 0.07)	0.14	0.128	0.980
D24‐25	FDM	6.46	0.21	0.94	−0.24 (−0.58, 0.17)	0.17	0.163	0.807
	DLP	6.73	0.15	0.67	−0.03 (−0.29, 0.24)	0.13	0.820	0.977
D25‐26	FDM	6.16	0.19	0.85	−0.05 (−0.30, 0.19)	0.12	0.652	0.888
	DLP	6.33	0.18	0.81	−0.12 (−0.39, 0.16)	0.13	0.387	0.960
G16‐14	FDM	14.29	0.46	2.07	−0.22 (−0.87, 0.43)	0.31	0.492	0.862
	DLP	14.50	0.40	1.79	0.00 (−0.37, 0.37)	0.18	0.980	0.889
G14‐11	FDM	27.55	0.47	2.09	−0.49 (−1.25, −0.26)	0.25	0.005	0.892
	DLP	28.16	0.47	2.12	0.50 (−0.09, 1.09)	0.28	0.092	0.910
G21‐24	FDM	26.79	0.62	2.78	−0.48 (−1.10, 0.13)	0.42	0.093	0.838
	DLP	27.42	0.65	2.92	0.11 (−0.86, 1.08)	0.46	0.812	0.901
G24‐26	FDM	15.31	0.36	1.61	0.06 (−0.34, 0.46)	0.19	0.766	0.912
	DLP	15.53	0.32	1.43	−0.29 (−0.78, 0.21)	0.24	0.243	0.925
U1ThickR	FDM	5.90	0.11	0.49	−0.12 (−0.21, −0.03)	0.04	0.015	0.946
	DLP	6.09	0.13	0.59	0.06 (−0.11, 0.24)	0.08	0.463	0.945
U1ThickL	FDM	5.80	0.13	0.59	−0.21 (−0.43, −0.00)	0.10	0.048	0.802
	DLP	6.12	0.14	0.64	0.01 (−0.33, 0.34)	0.16	0.973	0.960

*Note*: Bonferroni's correction was used to adjust the level of significance to 0.002. Variables' definitions are given in Table 1.

^a^ Systemic error was assessed using paired *t* tests.

^b^ Random error was assessed using Intra class Correlation Coefficient based on absolute agreement.

Abbreviations: ICC, Intra class Correlation Coefficient; SD, Standard Deviation; SE, Standard Error.

**TABLE 4:** Descriptive statistics of the measurements made on the three sets of models (*n* = 20) as well as the mean differences between the two types of printed models and the gold standard plaster models in conjunction with the *p*‐values of statistical testing and the intra‐class correlation coefficients

Measurements	Type	Mean	SE	SD	Mean difference (95% CI)	SE mean difference	*p*‐value^a^	ICC^b^
LCCW	FDM	26.04	0.50	2.24	−0.36 (−0.81, 0.17)	0.25	0.173	0.931
	DLP	26.33	0.49	2.17	0.07 (−0.39, 0.54)	0.22	0.739	0.945
LPPM	FDM	33.55	0.57	2.55	−0.25 (−1.34, 0.83)	0.52	0.632	0.954
	DLP	33.80	0.50	2.25	0.00 (−1.01, 1.01)	0.48	0.993	0.965
LMMW	FDM	42.88	0.60	2.69	0.17 (−0.68, 1.02)	0.41	0.684	0.880
	DLP	43.13	0.57	2.55	−0.42 (−1.51, 0.42)	0.40	0.309	0.889
D36‐35	FDM	6.32	0.17	0.74	−0.18 (−0.32, −0.03)	0.07	0.017	0.969
	DLP	6.50	0.18	0.80	0.16 (−0.08, 0.39)	0.11	0.174	0.933
D35‐34	FDM	6.93	0.22	0.99	−0.11 (−0.36, 0.14)	0.12	0.371	0.911
	DLP	7.32	0.25	1.11	−0.24 (−0.52, −0.04)	0.12	0.023	0.955
D34‐33	FDM	7.16	0.20	0.88	−0.23 (−0.50, −0.04)	0.12	0.038	0.903
	DLP	7.58	0.15	0.67	−0.12 (−0.35, 0.11)	0.11	0.292	0.930
D33‐32	FDM	7.01	0.21	0.94	−0.12 (−0.35, 0.12)	0.11	0.318	0.897
	DLP	6.95	0.18	0.81	0.18 (0.025, 0.33)	0.07	0.025	0.901
D32‐31	FDM	5.09	0.16	0.71	0.09 (−0.12, 0.29)	0.10	0.393	0.890
	DLP	5.17	0.18	0.79	−0.16 (−0.35, 0.03)	0.09	0.100	0.866
D31‐41	FDM	4.50	0.13	0.57	0.02 (−0.16, 0.20)	0.09	0.839	0.836
	DLP	4.57	0.12	0.55	−0.09 (−0.25, 0.07)	0.08	0.272	0.965
D41‐42	FDM	5.05	0.20	0.89	0.13 (−0.17, 0.42)	0.14	0.390	0.809
	DLP	5.09	0.18	0.81	−0.17 (−0.47, 0.14)	0.15	0.274	0.941
D42‐43	FDM	6.42	0.27	1.21	−0.19 (−0.43, 0.05)	0.12	0.118	0.928
	DLP	6.73	0.25	1.13	0.15 (−0.05, 0.34)	0.09	0.136	0.938
D43‐44	FDM	7.29	0.21	0.93	−0.13 (−0.37, 0.12)	0.12	0.300	0.898
	DLP	7.50	0.21	0.95	−0.08 (−0.30, 0.14)	0.11	0.468	0.888
D44‐45	FDM	6.76	0.23	1.02	−0.13 (−0.33, 0.08)	0.10	0.211	0.957
	DLP	7.25	0.27	1.19	0.05 (−0.11, 0.21)	0.08	0.529	0.935
D45‐46	FDM	6.26	0.21	0.93	−0.09 (−0.28, 0.10)	0.09	0.339	0.895
	DLP	6.44	0.23	1.00	0.17 (−0.13, 0.46)	0.14	0.254	0.954
G36‐34	FDM	16.74	0.36	1.61	−0.01 (−0.39, 0.38)	0.19	0.979	0.933
	DLP	16.86	0.37	1.64	−0.12 (−0.50, 0.26)	0.18	0.520	0.990
G34‐31	FDM	23.21	0.44	1.95	−0.49 (−1.01, −0.03)	0.22	0.022	0.912
	DLP	23.53	0.46	2.07	0.23 (−0.28, 0.68)	0.21	0.294	0.901
G41‐44	FDM	22.69	0.44	1.98	−0.49 (−0.76, −0.22)	0.13	0.003	0.966
	DLP	23.20	0.45	2.02	0.08 (−0.18, 0.34)	0.13	0.541	0.933
G44‐46	FDM	16.10	0.39	1.76	0.13 (−0.27, 0.52)	0.19	0.509	0.945
	DLP	16.36	0.41	1.85	−0.38 (−0.86, 0.09)	0.23	0.110	0.954
L1ThickL	FDM	5.39	0.15	0.69	−0.10 (−0.30, 0.10)	0.10	0.309	0.823
	DLP	5.61	0.15	0.66	0.13 (−0.13, 0.38)	0.12	0.321	0.853
L1ThickR	FDM	5.24	0.11	0.49	−0.24 (−0.35, −0.18)	0.05	0.009	0.869
	DLP	5.42	0.14	0.60	0.17 (0.01, 0.33)	0.08	0.041	0.990

*Note*: Bonferroni's correction was used to adjust the level of significance to 0.002. Variables' definitions are given in Table 2.

^a^ Systemic error was assessed using paired *t* tests.

^b^ Random error was assessed using Intra class Correlation Coefficient based on absolute agreement.

Abbreviations: ICC, Intra class Correlation Coefficient; SD, Standard Deviation; SE, Standard Error.

### 3.1 FDM models versus plaster models

ICCs ranged from 0.802 to 0.990 indicating excellent agreement between the FDM and plaster models. There were no significant differences in the measurements made between the FDM printed models and the original plaster models (*p* ˃ 0.002), with an overall mean difference of −0.11 mm (range: from −0.49 to 0.17 mm). For the upper models, the average mean differences of the intra‐arch width, inter‐dental, within‐gingival, and tooth thickness measurements were −0.01, −0.07, −0.28, and −0.17 mm respectively. For the lower models, the average mean difference of the intra‐arch width, inter‐dental, within‐gingival, and tooth thickness measurements were −0.15, −0.06, −0.22, and −0.17 mm respectively.

### 3.2 DLP models versus plaster models

ICCs ranged from 0.853 to 0.990 indicating excellent agreement between the DLP and plaster models. There were no significant differences in the measurements made between DLP printed models and the original plaster models (*p* ˃ 0.002) with an overall mean difference of 0.00 mm (range: from −0.42 to 0.50 mm). For the upper models, the average mean differences of the intra‐arch width, inter‐dental, within‐gingival, and tooth thickness measurements were 0.31, −0.09, 0.08, and 0.04 mm respectively. For the lower models, the average mean differences in the intra‐arch width, inter‐dental, within‐gingival, and tooth thickness measurements were −0.12, −0.01, −0.05, and 0.15 mm respectively.

## 4 DISCUSSION

Although the digital scanning process has been accepted as an accurate step in the digital workflow, the 3D printing process, which is another important step, still needs more investigations. Several studies have defined or established a range of error for linear measurements that have been considered clinically acceptable when replicating the plaster models or printing the digital models for diagnostic purposes. According to the ABO standards for grading plaster models, an intra‐arch distance with an error less than 0.5 mm from the gold‐standard plaster model is considered clinically accepted. Rebong et al. reported that a difference in dimensions between model types equal to or less than 0.5 mm was unlikely to have a significant clinical impact (Rebong et al., 2018). Many studies (Halazonetis, 2001; Hassan et al., 2017; Hazeveld et al., 2014; Schirmer & Wiltshire, 1997) have reported that the range between 0.2 and 0.5 mm is considered as an acceptable range for clinical accuracy.

However, these clinical standards for dental model accuracy should be stricter when it comes to the fabrication of clear aligners on printed 3D models. Clear aligner therapy consists of several aligners with an average transitional movement of 0.25–0.30 mm for each tooth in each consequent appliance (Vlaskalic & Boyd, 2001). Thus, the difference in the measurement accuracy between the printed models and their originals must be smaller than 0.25–0.30 mm to enable the fabricated aligner exert a proper orthodontic force on the targeted teeth. Therefore, when the printed models are aimed to be used for clear aligners fabrication, a 0.25‐mm dimensional mean discrepancy should be considered clinically acceptable for the inter‐dental and tooth thickness measurements, whereas a 0.5‐mm dimensional mean discrepancy should be acceptable for the intra‐arch and within‐gingival measurements due to their relatively large values (measurements were made between landmarks placed distant from each other).

Additionally, when considering the ability of the researcher to repeatedly identify several landmarks on plaster or printed models, there would be an amount of identification error that could affect the evaluation procedure of the 3D printed models (Bell et al., 2003; Houston, 1983). To eliminate this error, 21 ball‐shaped reference points were placed on the original plaster models after scanning and printing their replicas. Therefore, the analysis of accuracy between measurements would allow the attribution of any observed differences to scanning and/or printing errors. However, the scanning procedure has been already validated and the error margin can be considered negligible (Kim et al., 2018).

There were no statistically significant differences between the models printed by either FDM or DLP techniques compared to the plaster models (*p* > 0.002). For the inter‐dental and tooth thickness measurements, all were smaller than the 0.25‐mm clinical threshold, and both techniques displayed similar variations from the gold standard models. Intra‐arch and within‐gingival measurements were also within the acceptance limit defined in this study (0.5 mm).

The results of this study demonstrated that FDM and DLP seemed to be valid to replace the orthodontic plaster models for analytic and diagnostic purposes. But if the printed models were produced to fabricate clear aligners, the amount of error observed in both printing techniques would lead probably to incomplete settling of clear aligners when tried in the mouth at their first application. Because of this amount of impreciseness, the use of “zero aligners” with no tooth movement in the preliminary stage of aligner treatment should always be encouraged to allow patients to get used to the new system as well as to get rid of any residual inadaptations due to the printing procedure.

Our study results are similar to the findings of Kasparova et al. (2013) who compared linear measurements between 10 plaster models and their FDM replicas and found that the mean differences for the intercanine width, the canine's clinical crown height, and the incisor‐canine distance were −0.17, −0.03, and 0.04 mm respectively. Consequently, they concluded that FDM models could replace the process of plaster making.

Murugesan et al. (2012), who fabricated dental models using three different types of 3D printers, reported that accuracy was the highest for the Polyjet technique (mean dimensional error of 0.133%), followed by the 3DP (powder‐based technique) and FDM techniques (mean dimensional error was 1.67% and 1.73%, respectively) in comparison to the gold‐standard virtual 3D stereolithographic models. The current findings are also consistent with those of Kim et al. (2018) who reported that the trueness of overall tooth measurements was higher for the Polyjet technique, followed by the SLA, DLP, and FDM techniques with mean root mean square (RMS) values of 78, 107, 143, and 188 μm, respectively. However, the current findings not agree with the those of Rebong et al. (2018) who reported that FDM models had the least differences from the original plaster models in comparison to the SLA and Polyjet techniques.

In the process of 3D printing, the printing materials may experience shrinkage and/or expansion during curing which may explain the increasing and decreasing tendencies of the printed models (Barker et al., 1994; Brown et al., 2018; Keating et al., 2008). In this study, the average of the mean difference between the plaster models and their FDM replicas was –0.11 mm which may be considered clinically negligible, whereas the average of the mean difference between the plaster models and their DLP replicas was 0 indicating that both techniques have dimensional stability during their construction. According to current results, the null hypothesis that postulated no significant differences in the measured dimensions between the printed models and the original plaster models cannot be rejected.

In the current investigation, some surface roughness was noticed in several 3D printed models. This roughness was accompanied by very tiny serrations and fluctuations, which may affect the final adaptation of the construed clear aligners when tried in the mouth. Future research work on 3D printed models should also focus on surface smoothness/roughness as well as dimensional accuracy. Other 3D printing technologies should also be evaluated in comparison with the commonly used techniques to enable the orthodontist/technician to choose the best method for clear plate fabrication.

## 5 CONCLUSIONS

- FDM and DLP models had no significant differences in comparison to the original models (*p* ˃ 0.002).
- Generally, The accuracy of the produced 3D models by the FDM and DLP techniques seemed acceptable.
- Using FDM and DLP printed models to fabricate clear aligners does not completely guarantee the optimum intraoral fit of the produced plates on the dental arches.

## CONFLICT OF INTEREST

The authors declare no conflicts of interest.

## AUTHOR CONTRIBUTIONS

Samer T Jaber made all the measurements, analyzed the collected data, interpreted the results and wrote the first drafts of this manuscript, Mohammad Y Hajeer supervised this work, planned the study design, helped in the statistical analysis, and help in writing up the manuscript. Tarek Z Khattab contributed to the study design and helped in data analysis. Luai Mahaini supervised the technical aspects of this research and helped in writing up the first drafts of this paper. All authors read and approved the final manuscript.

## ETHICS STATEMENT

Ethics approval and consent to participate: Ethical Approval was obtained from the Research Ethics Committee of University of Hamah Dental School, Syria (Approval no. UHDS‐5104_2019PG).

## Data Availability

The data that support the findings of this study are available from the corresponding author upon reasonable request.
